# Association between the Expression Levels of MicroRNA-101, -103, and -29a with Autotaxin and Lysophosphatidic Acid Receptor 2 Expression in Gastric Cancer Patients

**DOI:** 10.1155/2022/8034038

**Published:** 2022-04-11

**Authors:** Sara Tutunchi, Saeedeh Akhavan, Ghodratollah Panahi, Mina Zare, Amirnader Emami Razavi, Reza Shirkoohi

**Affiliations:** ^1^Department of Medical Genetics, Shahid Sadoughi University of Medical Sciences, Yazd, Iran Postal Code: 8915173143; ^2^Department of Biology, School of Basic Sciences, Science and Research Branch, Islamic Azad University (IAU), Tehran, Iran Postal Code: 1477893855; ^3^Department of Biochemistry, Faculty of Medicine, Tehran University of Medical Sciences, Tehran, Iran Postal Code: 1417653761; ^4^Recombinant Protein Laboratory, Department of Biochemistry, Shiraz University of Medical Sciences, Shiraz, Iran Postal Code: 7134845794; ^5^Iran National Tumor Bank, Cancer Institute of Iran, Tehran University of Medical Sciences, Tehran, Iran Postal Code: 1419733141; ^6^Cancer Biology Research Center, Cancer Research Institute, Imam Khomeini Hospital Complex, Tehran University of Medical Sciences, Tehran, Iran Postal Code: 1419733141

## Abstract

**Background:**

Gastric cancer (GC) is regarded as the most prevalent malignancy with the high mortality rate, worldwide. However, gastroscopy, a biopsy of suspected sample, and detecting CEA, CA19-9, and CA72-4 are presently used, but these diagnostic approaches have several limitations. Recently, microRNAs as the most important member of noncoding RNAs (ncRNAs) have received attention; recent evidence demonstrates that they can be used as the promising candidate biomarkers for GC diagnosis. We aimed to investigate the association between the microRNA-29a, -101, and -103 expression and autotaxin (ATX) and lysophosphatidic acid receptor 2 (LPA2) expression in GC patients. *Material and Methods*. The present study was conducted on 40 paired samples of primary GC tissue and adjacent noncancerous tissue. The gene expression levels of miR-101, -103, -29, ATX, and LPA2 were analyzed by quantitative reverse-transcription PCR (qRT-PCR). Besides, the protein levels of ATX and LPA2 were evaluated using western blot.

**Results:**

The expression levels of miR-29 and miR-101 were significantly lower (*p* value < 0.0001), but the miR-103 and LPA2 were significantly higher in gastric tumor samples compared to the corresponding nontumor tissues (*p* value < 0.0001). Moreover, the diagnostic accuracy of miRs to discrimine the GC patients from noncancerous controls was reliable (miR-101, sensitivity: 82.5% and specificity: 85%; miR-103, sensitivity: 72.5% and specificity: 90%; miR-29, sensitivity: 77.5% and specificity: 70%).

**Conclusion:**

It seems that determining the expression level of miR-101, -103, and -29, as the novel diagnostic biomarkers, has diagnostic value to distinguish GC patients from healthy individuals.

## 1. Introduction

Gastric cancer (GC) is a heterogeneous disease. It is known as the fourth most prevalent cancer in men and the fifth in women; it has become the second leading cause of cancer-related death worldwide [1, 2]. Recently, it was found that the GC incidence in the western Asia countries is high. In Iran, GC has an annual incidence of 7300, which is the first cause of cancer-related death in both genders. Also, the 5-year survival rate is reported to be less than 25% [3]. GC is only diagnosed in the early stages in less than 10% of cases, so that late diagnosis may lead to disease progression and reduced survival rate. The high mortality and morbidity rates are due to the late diagnosis, unfavorable prognosis, and resistance to treatment [4–6].

Gastroscopy is considered as the golden standard method for GC diagnosis, but the false-negative rate results require looking for other strategies [7–9]. Nowadays, diverse molecules have been evaluated as promising biomarkers in GC patients. For instance, carcinoembryonic antigen (CEA) and cancer antigen 19-9 (CA19-9) are introduced as diagnostic biomarkers, which in turn are used for GC screening; they have low sensitivity, especially at the early stages [10–12]. Hence, the identification of a high sensitive noninvasive method is necessary.

MicroRNAs (miRNAs), as the most famous noncoding RNAs (ncRNAs), are implicated in different biological events. These multitasking molecules are epigenetics modulators, which regulate gene expression at the posttranscriptional levels [13–15]. Recently, compelling evidence introduced miRNAs as new tools to diagnosis different cancers including breast, lung, colorectal, esophageal, and GC at the early stage [16–18]. More importantly, miRNAs are detectable in different biological body fluids, including serum, urine, saliva, CSF, and semen; they are resistant to nucleolytic degradation and are stable against environmental conditions such as freeze and thaw [19, 20]. Hence, these small molecules can be regarded as noninvasive diagnostic biomarkers at the early stage of various cancers, mainly GC.

It was demonstrated that the circulating miRNA-17 and miRNA-25 were remarkably elevated in the serum sample of GC patients compared to the healthy individuals; this finding suggests the cited miRNAs as the diagnostic biomarkers for the early detection of GC [21].

Lysophosphatidic acid (LPA) as a simple lipid consists of an acyl chain of a glycerol backbone; it activates six G-coupled protein receptors (LPA1–6) at least, inducing various signaling pathways [22]. There is evidence that LPA2 is involved in proliferation, apoptosis, drug resistance, metastasis, and the invasion of numerous cancer cells, as well as expression increment in different tumor tissues, including breast, liver, gastric, and colorectal cancers [23–26].

LPA is generated by the autotoxin (ATX) hydrolysis of lysophosphatidylcholine. ATX is an important extracellular lysophospholipase D enzyme that is overexpressed in the renal cell carcinoma, breast and ovarian cancer, and bladder carcinoma [27, 28]. There is increasing data, showing miRNAs regulate ATX and LPA expression [29, 30]. It has been reported that miR-101-3p suppresses ATX expression; also, an inverse correlation was found between ATX and miR-101-3p levels in U87 (astrocytoma cell line) and HCT116 (colorectal carcinoma cell line) cancer cells [31]. Furthermore, it was illustrated that the ATX signaling axis was reduced in chronic obstructive pulmonary disease (COPD) patients. This reduction corresponded to an increase in miR-29 family members [32]. Respecting the crucial roles of ATX and LPA2 in cancer progression, evaluating specific miRNAs, which control ATX and LPA2 expression, can be helpful in the development of novel therapeutic strategies for GC patients.

The present study is aimed at investigating the serum levels of miRNA-101, -103, and -29a in GC patients, their correlation with clinicopathological characteristics, and ATX and LPA2 expression; it may help to find a new strategy in the diagnosis and treatment of GC patients.

## 2. Material and Methods

### 2.1. Study Population

Forty paired samples of primary GC tissue and adjacent noncancerous tissue (>3 cm from the tumor tissue) were collected from patients; they had been recruited during April 2019-July 2020 at the Iran National Tumor Bank, Cancer Institute, Imam Khomeini Hospital in Tehran, Iran. GC diagnosis was approved based on the clinical examination and histopathology tests conducted by an expert pathologist.

Exclusion criteria of the present study were as follows: patients with prior history of other malignancies, infection diseases, coagulation disorders, mental illness, or psychosis and those who had undergone chemotherapy, radiotherapy, and targeted therapy. All collected tissues, including GC and adjacent noncancerous tissues after removal from patients' bodies, were frozen and stored at -70 in liquid nitrogen until the final analysis. Also, clinicopathological characteristics of all patients were documented from the clinical and pathological records (Table 1). This study was confirmed by the Ethics Committee at Tehran University of Medical Sciences (IR.TUMS.MEDICINE.REC.1399.1087). All patients provided written informed consent through the Iran National Tumor bank.

### 2.2. RNA Extraction and cDNA Synthesis

Total RNA from tissues was extracted with Total RNA Purification Kit (Norgen Biotek, Canada) following the manufacturer's protocol. The quality and quantity of each isolated RNA were determined by OD260/280 using a NanoDrop spectrophotometer (Thermo Fisher Scientific). Afterward, for the cDNA synthesis of miRNA-101, -103, and -29a, purified RNA samples were polyadenylated by poly (A) polymerase; then, they were reverse transcribed to cDNA using a mixture of oligo (dT) adaptor provided in the kit (microScript microRNA cDNA Synthesis Kit, Norgen Biotek, Canada). Besides, cDNA synthesis of LPA2 and ATX was done by using 2 *μ*g of total RNA and 1 *μ*L of random hexamers as well as oligo (dT), 5× first-strand buffer, 1 *μ*L dNTPs, and 0.5 *μ*L RNasin and M-MLV for each sample.

### 2.3. Quantitative Reverse-Transcription PCR (qRT-PCR)

qRT-PCR was done using SYBR Premix Ex Taq II (Takara, Dalian, Liaoning, China) to detect the miRNA-101, -103, and -29a, ATX, and LRP2 expression as target genes; the *β*-actin was used as housekeeping gene for normalization. The real-time PCR cycler RotorGene Q (QIAGEN GmbH, Hilden, Germany) was used to perform the reactions. All reactions were performed in triplicate. Furthermore, the relative expression of miRNA-101, -103, -29a, ATX, and LRP2 primary GC tissue and adjacent noncancerous tissue was evaluated using the comparative cycle threshold (Ct) (2-*ΔΔ*Ct) method. The detailed sequences of primers for amplifying miRNA-101, -103, and -29a, ATX, and LRP2 have been presented in Table I of the supplementary file I.

### 2.4. Western Blot Analysis

Total protein was extracted from homogenized GC and adjacent noncancerous tissues; it was done using modified radioimmunoprecipitation (RIPA) buffer. Then, equal amounts of proteins were resolved by 10% sodium dodecyl sulfate-polyacrylamide gel electrophoresis (SDS-PAGE) and transferred onto a PVDF membrane. Afterward, membranes were incubated in 5% nonfat milk or bovine serum albumin (BSA) for 2 hours at room temperature and then incubated for 24 hours at 4C° with LPA2, ATX, and GAPDH primary antibodies. Next, they were exposed to the secondary antibody for 1 hour. Finally, labeled proteins were visualized using enhanced chemiluminescent substrate (ECL, Amersham). The bands intensity was assessed by densitometry via the ImageJ software.

## 3. Results

### 3.1. Evaluation of miR-101, -103, and -29 Expression in GC Tissues and Adjacent Nonneoplastic Tissues

**Figure 1:**
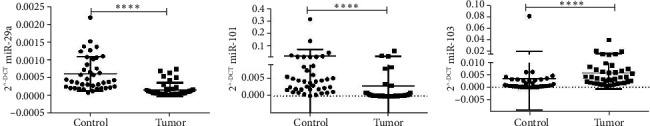
Expression levels of miR-29 (a), miR-101 (b), and miR-103 (c) in GC tissues relative to the adjacent noncancerous tissues.

As illustrated in Figure 1, the RT-qPCR findings demonstrated that the expression levels of miR-29 and miR-101 were significantly lower in GC specimens in comparison to the adjacent noncancerous tissues (*p* value < 0.0001). However, the miR-103 was overexpressed in gastric tumor samples compared to the corresponding nontumor tissues (*p* value < 0.0001) (Figure 1).

### 3.2. Comparison of ATX and LPA2 Expression in GC and Adjacent Nonneoplastic Tissues

**Figure 2:**
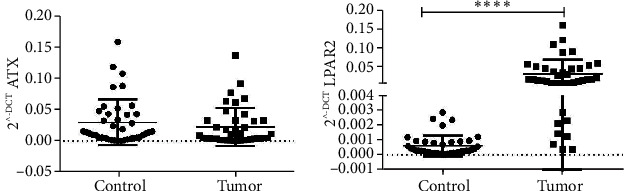
Expression levels of ATX (a) and LPA2 (b) in GC tissues relative to the adjacent noncancerous tissues.

The ATX and LPA2 expression was measured in the GC and noncancerous tissues. As presented in Figure 2, there was no any significant changes in the expression levels of ATX in GC tissues in comparison to the noncancerous tissues (*p* value = 0.2412). We observed that LPA2 expression was significantly higher in the GC samples (*p* value < 0.0001) (Figure 2).

### 3.3. Determination the ATX and LPA2 Protein Expression of GC and Adjacent Nonneoplastic Tissues

**Figure 3:**
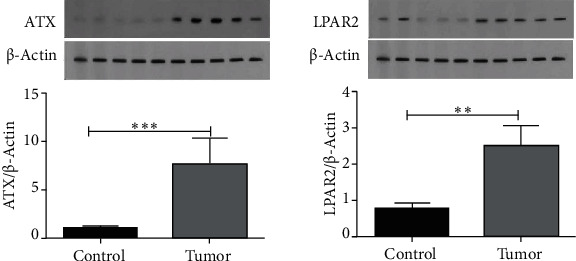
Protein levels of ATX (a) and LPA2 (b) in GC tissues relative to the adjacent noncancerous tissues.

The protein expression levels of ATX were significantly higher in GC tissues (*p* value < 0.0002). Similarly, our data revealed a remarkable increase in protein levels of LPA2 in GC specimens compared to the noncancerous controls (*p* value < 0.0001) (Figure 3).

### 3.4. Correlation between the Gene Expression Levels of miRs, ATX, and LPA2 with Clinicopathological Properties

We next assessed the correlation of miR-103, miR-29, miR-101, ATX, and LPA2 expression with clinicopathological properties, including sex, site of primary, necrosis presence, vascular invasion, perineural invasion, TNM staging, tumor size, pathological grade, and family history in GC tissues. According to the findings presented in Table 2, there was no significant correlation between the expression of miRs, ATX, and LPA2 with none of the clinicopathological characterizations.

### 3.5. Assessment of miR-101, -103, and -29 as Promising Candidate Biomarkers for GC Detection

**Figure 4:**
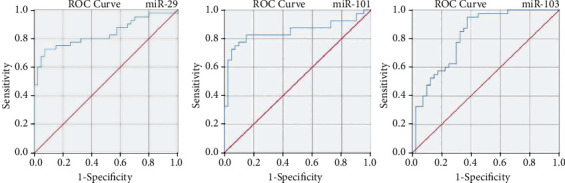
Potential diagnostic accuracy of tissue miR-29, -101, and -103 to discriminate GC patients from adjacent noncancerous tissues.

To investigate whether the expression levels of miR-101, -103, and -29 have diagnostic value to discriminate GC patients from healthy individuals, the receiver operating characteristic (ROC) curves was depicted, and the area under the curve (AUC) values were computed. Given the ROC results, miR-101 (sensitivity: 82.5 and specificity: 85%), miR-103 (sensitivity: 72.5 and specificity: 90%), and miR-29 (sensitivity: 77.5 and specificity: 70%) with suitable sensitivity, specificity, and reasonable AUC had diagnostic accuracy for GC detection, as illustrated in Figure 4.

## 4. Discussion

More recently, clinicians have focused on various invasive and noninvasive biomarkers to diagnose GC patients at the early stage. Gastroscopy, a biopsy of suspected samples, and detecting the serum biochemical tumor markers such as CEA, CA19-9, and CA72-4 are presently utilized. However, these diagnostic strategies have proven to be helpful, but they have some limitations. Hence, exploring noninvasive biomarkers with high sensitivity and specificity can help physicians for detecting the GC patients.

Emerging evidence elucidated that several miRNAs can act as diagnostic biomarkers in GC; they are measurable in various body fluids such as serum, urine, gastric juice, and CSF [33]. Also, regarding their resistance to RNases, boiling, pH changes, and extended storage [34], they can be beneficial markers for GC diagnosis. Our results exhibited that expression levels of miR-29 and miR-101 significantly reduced, whereas miR-103 and LPA2 expression remarkably increased in GC tissues compared to the corresponding noncancerous tissues. Although, the gene expression level of ATX was not altered between the GC and normal tissues.

Interestingly, compared with adjacent nontumor groups, the protein level of ATX and LPA2 was substantially higher in GC specimens. It has been reported that miR-29 as a tumor-suppressive miRNA is downregulated in different cancers, including osteosarcoma, glioblastoma, hepatocellular carcinoma, bladder, prostate, renal, lung, and ovarian cancers [35, 36]. Following our results, several studies revealed that the expression level of miR-29 was elevated in GC tissues compared to the normal tissues; they also suggested that the miR expression level can be possibly employed as a new diagnostic biomarker in GC patients [37].

On the other hand, there are controversial data about expression levels of miR-101 in various cancers. However, most reports demonstrated that this miRNA is a tumor suppressor gene with lower expression in diverse cancers [38, 39]. Two studies reported its upregulation in prostate cancer tissue HepG2 cells [40, 41]. Recently, Dong and Liu found that the expression level of miR-101 was reduced in GC tissues. It correlated with the pathological differentiation degree of the tumor, lymph node metastasis, and depth of infiltration [42]. In addition, similar to our results, several studies reported that this miRNA reduced in GC samples in comparison to the cancer-adjacent normal tissues [43, 44].

Another important miRNA in the pathogenesis of GC is miR-103, which is considered oncogenic in different types of cancers [45, 46]. It has been found that miR-103 is overexpressed in the animal model of diffuse-type GC [47]. There are conflicting reports regarding tissue expression levels of miR-103 in different types of GC patients. So, Parvaee and colleagues exhibited that miR-103 expression was not significantly changed between the intestinal-type of GC compared to the healthy individuals [48].

Moreover, in another study, the observed plasma expression of miR-103 in different GC cases with or without any intestinal components was not remarkable. This miRNA was not appropriate for diffuse-type GC screening [49]. By contrast, similar to our findings, it was elucidated that the expression level of miR-103 was elevated in advanced GC tissues in comparison to the normal gastric mucosa of cancer-free subjects [50]. It seems that sample type is the reason for these controversial reports.

Many in vitro and in vivo researches have revealed elevated LPA/ATX signaling participates in cancer initiation and development. LPA2, as a G protein-coupled receptor, is extensively expressed in several tumors, including breast, ovarian, thyroid, and colorectal cancers [23]. This study observed that LPA2 gene and protein expression was enhanced in tumoral tissues toward adjacent nontumor tissues, which align with Yamashita's study [26]. They reported LPA2 elevation in intestinal-type GC; its expression is linked to an increased rate of lymphatic and venous invasion metastasis. Due to the fact that LPA2 is involved in the pathogenesis of GC, its inhibition by drugs can be a good treatment for patients.

ATX with lysophospholipase D activity implicates in LPA signaling pathway. This innovative molecule has crucial functions in angiogenesis, invasion, and metastasis. There is accumulating data that increased ATX has been detected in malignancies such as breast, pancreatic, non-small-cell lung, colorectal, and thyroid cancers [51–53]. In following with our findings, it has been demonstrated that serum activity of ATX enhanced in pancreatic cancer patients but not in GC ones [54, 55]. Nonetheless, related to the ATX protein, it was higher in GC tissue samples than the paired nontumor tissue specimens.

It seems, that miR-103, miR-29, and miR-101 with suitable sensitivity and specificity have good diagnostic performance for distinguishing GC patients from normal individuals. Several studies have shown that numerous miRNAs are reliable markers for diagnosis, prognosis, and clinical applications in GC at the early stages. In this way, following observation, Wang and coworkers reported that the cut of value 0.69 miR-29 fold change with 70% sensitivity and 68% specificity has appropriate diagnostic accuracy in the tumor tissue [56, 57]. Also, despite disagreements about using miR-103 as a diagnostic biomarker in GC, we found this miRNA helpful to detect GC patients with sensible AUC, sensitivity, and specificity. This disagreement probably is due to the sample size, inclusion and exclusion criteria, individual characterization, and analytical procedures.

Besides, miR-101 has been introduced as a diagnostic biomarker for GC discrimination by two studies [38, 58]. The clinical utility of serum miRNAs as promising diagnostic and prognostic biomarkers for GC needs more explorations. Indeed, the therapeutic applications of miRNAs as diagnostic and prognostic biomarkers for GC are not still completely determined, and further studies are necessary.

## 5. Conclusion

In summary, our result implied that gene expression levels of miR-101, -29, -103, and LPA2 increased in GC tissues toward adjacent noncancerous tissues. Moreover, compared to matched nontumor tissues, the protein status of LPA2 and ATX was higher in GC tissue samples. Meanwhile, all three miRNAs had diagnostic value with reliable AUC, sensitivity, and specificity to distinguish GC patients from healthy subjects.

### 5.1. Limitation Study

In future studies, it is better to investigate the relationship between miRs expression and patients' response to treatment. It is also necessary to evaluate the effect of different drugs, to select the most appropriate ones to regulate the expression of these genes, and ultimately increase patient survival.

## Figures and Tables

**Table 1:** Characteristics of included GC patients.

Clinical parameters		*N* (%)
Gender	Male	34 (85)
	Female	6 (15)
Site of primary	Cardian	5 (12.5)
	Antrum	9 (22.5)
	Body	26 (65)
Necrosis invasion	Yes	7 (16.6)
	No	20 (50)
	Unknown	13 (32.4)
Vascular invasion	Yes	22 (55)
	No	17 (42.4)
	Unknown	1 (2.6)
Perineural invasion	Yes	19 (47.6)
	No	19 (47.6)
	Unknown	2 (4.8)
Tumor size	<5	19 (47.6)
	≥5	21 (52.4)
Histological grading	I + II	22 (55)
	III + IV + V	18 (45)
TNM staging	I + II	19 (47.5)
	III + IV	21 (52.5)
Pathological N	N0	15 (37.5)
	N1	17 (42.5)
	N2	7 (17.5)
	N3	1 (2.5)
Pathological M	M0	37 (92.5)
	M1	3 (7.5)
Pathological T	T1 + T2	15 (37.5)
	T3 + T4	25 (62.5)
Family history	Yes	13 (32.5)
	No	27 (67.5)

**Table 2:** Comparison of diagnostic accuracy of microRNAs by ROC analysis for discrimination of the GC tissues of patients than adjacent noncancerous tissues.

MicroRNAs	AUC (CI)	Sensitivity (%)	Specificity (%)	*p* value
miR-101	0.849 (0.755-0.942)	82.5	85	<0.001
miR-29	0.834 (0.740-0.927)	72.5	90	<0.001
miR-103	0.819 (0.726-0.912)	77.5	70	<0.001

AUC: area under the curve; CI: confidence interval.

## Data Availability

The datasets used and analyzed during the current study are available from the corresponding author on reasonable request.

## Supplementary Materials

Supplementary Materials Table I: SeqTences of primers for qRT-PCR.
