# Supplementary material for: Association between the Expression Levels of MicroRNA-101, -103, and -29a with Autotaxin and Lysophosphatidic Acid Receptor 2 Expression in Gastric Cancer Patients
Source: J Oncol. 2022 Apr 11;2022:8034038. doi: 10.1155/2022/8034038 (PMC9015865; doi:10.1155/2022/8034038)
Supplement: Supplementary Materials — Table I: SeqTences of primers for qRT-PCR. [file 8034038.f1.docx]

**Table I.** SeqTences of Primers for qRT-PCR

| **Gene name** | **Forward primer**  SeqTence (5'->3') | **Reverse primer**  SeqTence (5'->3') |
| --- | --- | --- |
| ATX (ENPP2) | ACTTTTGCCGTTGGAGTCAAT | GGAGTCTGATAGCACTGTAGGA |
| LPAR2 | ACAGCCCGACTTTCACTTGAG | GCCCACAATGAGCATGACCA |
| β- Actin | CATGTACGTTGCTATCCAGGC | CTCCTTAATGTCACGCACGAT |
| miR-29a | TAGCACCATCTGAAATCGGTTA | universal |
| miR-101 | TACAGTACTGTGATAACTGAA | universal |
| miR-103 | AGCAGCATTGTACAGGGCTATGA | universal |
| T6 | CTCGCTTCGGCAGCACATATACT | universal |
